# Information within residency monthly evaluation forms at two institutions

**DOI:** 10.1080/10872981.2019.1635844

**Published:** 2019-06-27

**Authors:** Christopher J. Cooper, Paulette Wehner, Cindy Dailey, Nanette O’Connor, James Kleshinski, Joseph I. Shapiro

**Affiliations:** a Department of Medicine, University of Toledo School of Medicine and Health Sciences, Toledo, USA; b Joan C. Edwards College of Medicine, Marshall University, Huntington, USA

**Keywords:** Correlation, network analysis, competencies, milestones, ACGME

## Abstract

Periodic review of resident performance is an important aspect of residency training. Amongst allopathic residency programs, it is expected that the performance of resident physicians which can be grouped based on the ACGME core competencies, be assessed so as to allow for effective feedback and continuous improvement. Review of monthly evaluation forms for residents in the core ACGME programs at Marshall University and the University of Toledo demonstrated a wide spread in the number of Likert questions that faculty were asked to complete. This number ranged from a low of 7 in Surgery to a high of 65 in Psychiatry (both Marshall Programs). Correlation and network analysis were performed on these data. High degrees of correlations were noted between answers to questions (controlled for each resident) on these forms at both institutions. In other words, although evaluation scores varied tremendously amongst the different residents in all the programs studied, scores addressing different competencies tended to be very similar for the same resident, especially in some of the programs which were studied. Network analysis suggested that there were clusters of questions that produced essentially the same answer for a given resident, and these clusters were bigger in some of the different residency program assessment forms. This seemed to be more the rule in the residency programs with large numbers of Likert questions. The authors suggest that reducing the number of monthly questions used to address the core competencies in some programs may be possible without substantial loss of information.

## Introduction

Evaluation of post graduate or resident performance has been occurring since the development of such programs in the early 20th century following the famous ‘Flexner’ report calling out for improvements in the education of physicians [1]. However, the modern assessment of resident performance across the spectrum of competencies [2] has become much more complex [3]. Although the six basic competencies (1. medical knowledge, 2. patient care and procedural skills, 3. interpersonal and communication skills, 4. professionalism, 5. system-based practice and 6. practice-based learning and improvement) form the root of most evaluation systems [2,4,5], specialty evaluations have been allowed to, if not encouraged to, expand to query along far more axes of evaluation. This is not to say that American Board of Medical Specialties (ABMS) residency review committees (RRC) have insisted on longer evaluation forms; in fact they have not. However, virtually all of these RRCs have increased the granularity of competency assessment, and incorporation into monthly evaluation forms would seem to logically follow. For example, on the Nephrology program website, more than 50 items of assessment have been detailed under the heading of ACGME core competencies [6]. As most residency evaluation forms have incorporated the concepts of milestones and PGY-specific evaluations, these forms which are filled out by faculty (as well as other learners and, in some cases, other members of the health care team) have become more complex.

In the current study, we attempted to evaluate the information present in the monthly residency evaluation forms used by core ACGME programs at the University of Toledo and Marshall University. Hypothesizing that there might be redundancy in some of these forms, especially those that asked what superficially seemed to be a large number of duplicative questions, we decided to perform analysis examining how much correlation the answer to the different queries (controlled for the specific resident) had with each other.

## Methods

Records of the monthly evaluations of all residents from the academic year 2015–16 from the core residency programs of pediatrics (PED), internal medicine (IM), Obstetrics and Gynecology (OB), family and community medicine (FM), psychiatry (Psych) and Surgery (Surg) were queried from a data base created and maintained with New Innovations^TM^ software. These monthly evaluations of all residents were obtained in a de-identified manner (CD for Marshall, JK for University of Toledo) and shared as Excel worksheets. These worksheets were converted into csv files for importation into the open source analysis package R. Both the University of Toledo and Marshall University IRB categorized the study as IRB exempt.

Correlation and network analysis were performed on only the Likert scale questions from each institution. As obstetrics and gynecology at UT used many different forms for different rotations, we did not attempt correlation or network analysis with this residency. In addition to the Likert scale questions, all of the residency evaluation forms had summative questions and allowed for the entry of free text comments. These fields were not analyzed in this paper.

Data analysis of these Likert questions was performed with R, [7] primarily using statistics functions present in the base package as well as a heatmap/network plot called heatmap.2 in the gplots package, [8] which allowed for Pearson correlations (r values) to be performed on a matrix of numerical data derived from answers to questions on the monthly evaluation form. In addition to restricting ourselves to Likert style questions, questions that were often unanswered (i.e. > 50%) on the forms were deleted from subsequent analysis. With some residency programs with high percentages of complete records (i.e., no missing values), we also performed principal component analysis using the princomp function of the stats package within R [7] .

Some of the residency programs provided data that was suitable for principal component analysis. Rather than try to substitute average values into records to replace missing values, we restricted this analysis to those programs where a large percentage of resident evaluations were complete for every question. These were restricted to Marshall where there was a paucity of resident-level specific Likert questions. As the Marshall Psychiatry program had very few complete records, we restricted the principal component analysis to Marshall IM, OB, PEDS, Surg and FM.

## Results

**Figure 1.**
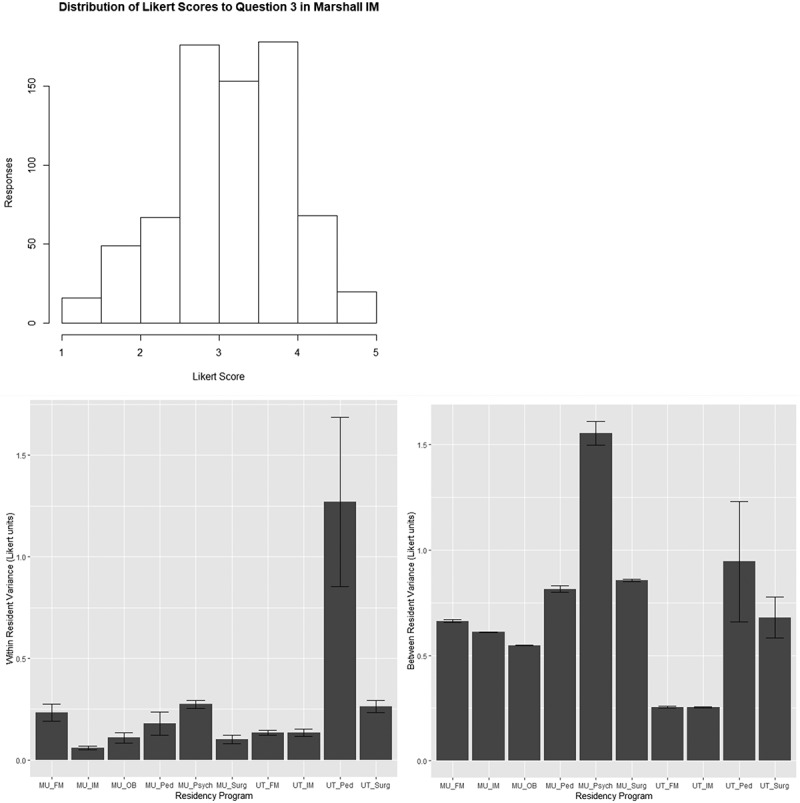
**Panel A: Histogram of resident scores in Marshall IM for question 3**. In contrast for the third subject in the Marshall IM program, all Likert scores were exactly 4. Panel B: Boxplot of Average Variance in Likert Score within each resident. Panel C: Boxplot of Average Variance in Likert Score across questions amongst (between) all residents. Data in Panels B and C shown as mean ± SD. Note that with one exception (UT Pediatrics), the average variance in Likert scores is greater between residents than within residents.

**Figure 2.**
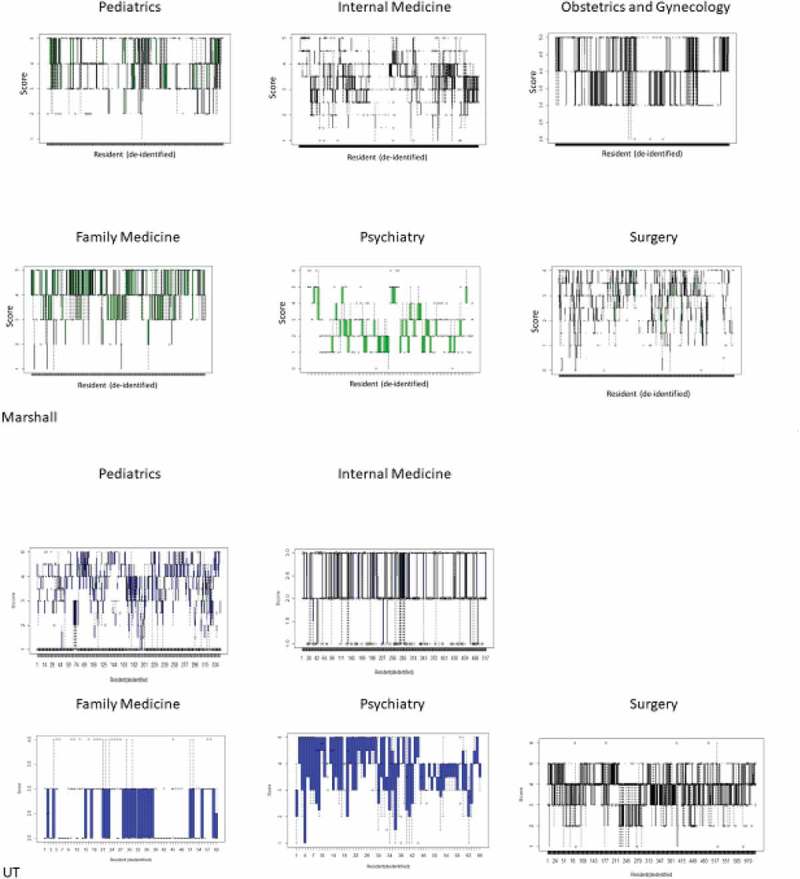
**Box plots showing distribution of scores per individual resident. Median marked with solid line, box traverses 1^st^ and 3^rd^ quartile**. Outlier values marked as solid circles. Data demonstrates the variability of scoring is greater between residents (residents numbered on x axis) than between Likert questions applied to the individual resident (y range) in somewhat different way that Figure 1 above.

Details for each residency program are provided in Table 1. We noted that variation in scores between residents varied considerably with all programs but that variations in the scores for specific residents on the different questions were quite similar. This is illustrated by the histogram detailing the responses to the third Marshall IM question that shows values across the answer scale. In contrast, looking at the scores on the 29 questions for the third resident in our list (no identification inherent in this), all the scores given were exactly 4 (Figure 1). The range of scores per de-identified resident are shown in Figure 2 and presented as boxplots.

**Table 1.** Basic information from Marshall and University of Toledo Programs.

	Marshall University		University of Toledo	
Program	Evaluations(N)	Likert Questions (N)	Evaluations (N)	Likert Questions (N)
Psychiatry	84	65	67	33
Surgery	491	7	642	15
Internal Medicine	728	29	520	26
Pediatrics	453	22	344	9
Obstetrics	742	16	109	22*
Family Medicine	372	9	61	30

University of Toledo Obstetrics used a number of different evaluation forms for different rotations and evaluators. Correlation analysis was not felt possible with the limited sample size for each form.

**Figure 3.**
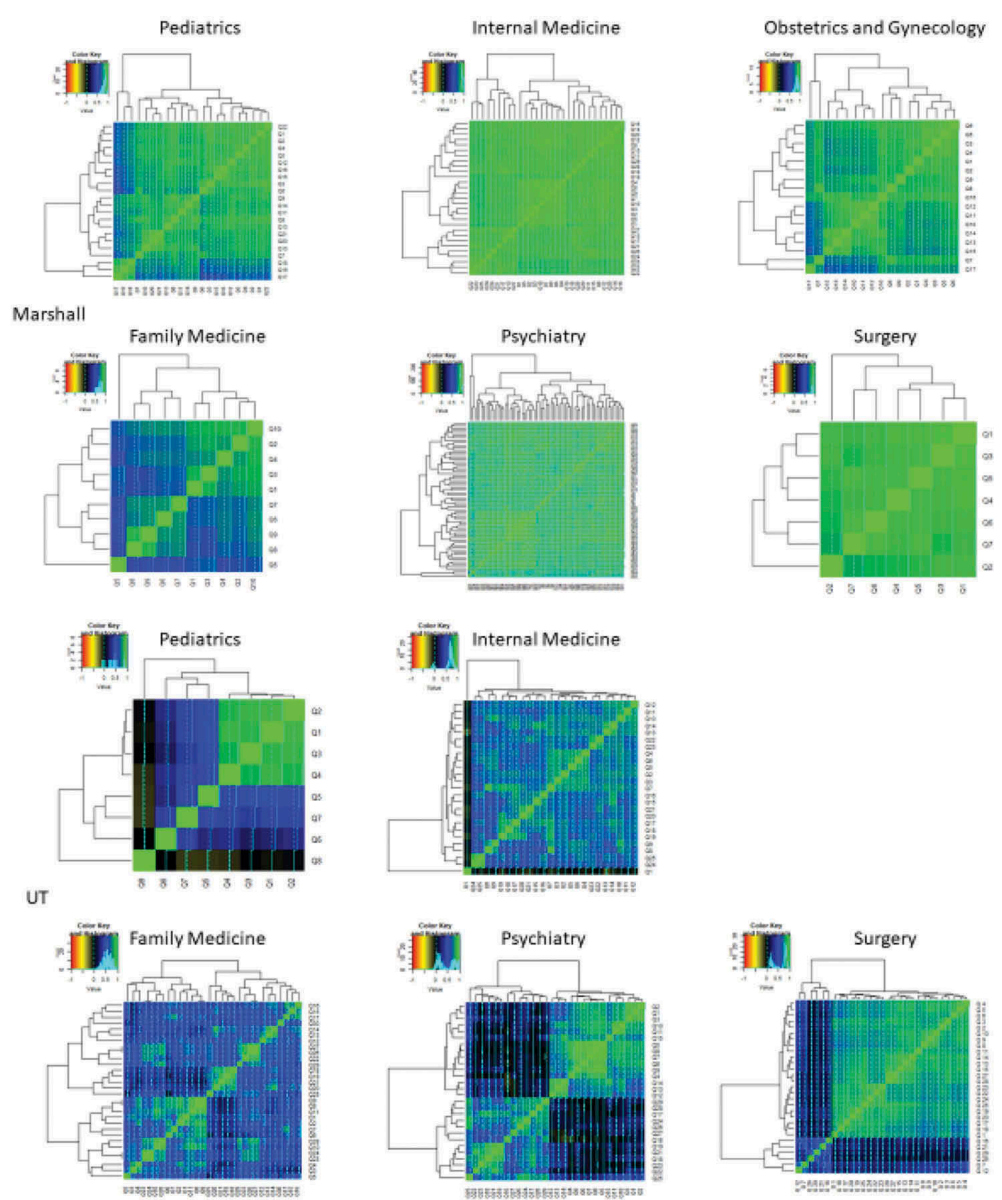
**Correlation heat maps with network groupings shown for Marshall (top Panel) and UT (bottom Panel) residency programs**. Color code for correlations shown on graphs. Network groupings fixed by R software using the command heatmap.2 within the package gplots. The results depict correlation between items with highly correlated items coded light green, as demonstrated by the line of identity for identical items that are displayed from bottom left to top right, to less well correlated items that are depicted in in darker shades.

Much of the Likert data correlated well within the resident evaluations, especially at the Marshall programs (Table 2, Figure 3). This is apparent even for those programs with the smallest number of questions such as Marshall FM and Surg. This can be seen on the correlation matrices shown in Table 2 where average Pearson correlation coefficients ranged from a low of 0.44 in the UT_Psych data set to average values exceeding 0.9 in Marshall Surg and IM.

**Table 2.** Correlations on forms from Marshall and University of Toledo Programs.

	Marshall University		University of Toledo	
Program	Mean	SD	Mean	SD
Psychiatry	0.88	0.04	0.45	0.34
Surgery	0.91	0.05	0.60	0.30
Internal Medicine	0.91	0.03	0.66	0.22
Pediatrics	0.83	0.08	0.46	0.37
Obstetrics	0.84	0.07		
Family Medicine	0.74	0.12	0.59	0.16

From these correlation values, network analysis was also performed. For Marshall PED, there were essentially three groups of highly correlated answers. For Marshall IM, the degree of correlation was so high as to make specific networks somewhat speculative. For Marshall OB, there were also three large grouping. In contrast, the answers to questions were only modestly correlated. Marshall Psych, like IM, had such highly correlated answers that groupings were difficult to visualize. Although Marshall Surgery had only 7 questions, the answers to all correlated quite well with each other. For the UT programs, the correlations were less high as previously noted. UT_PED showed a very highly correlated group of 4 questions with much less correlation noted between the remaining questions. UT_IM had a number of pockets of highly correlated answers as did UT_FM and UT_Psychiatry. UT Surgery had a large group of highly correlated questions with a subset (approximately 15%) of questions that correlated poorly with all others.

**Figure 4.**
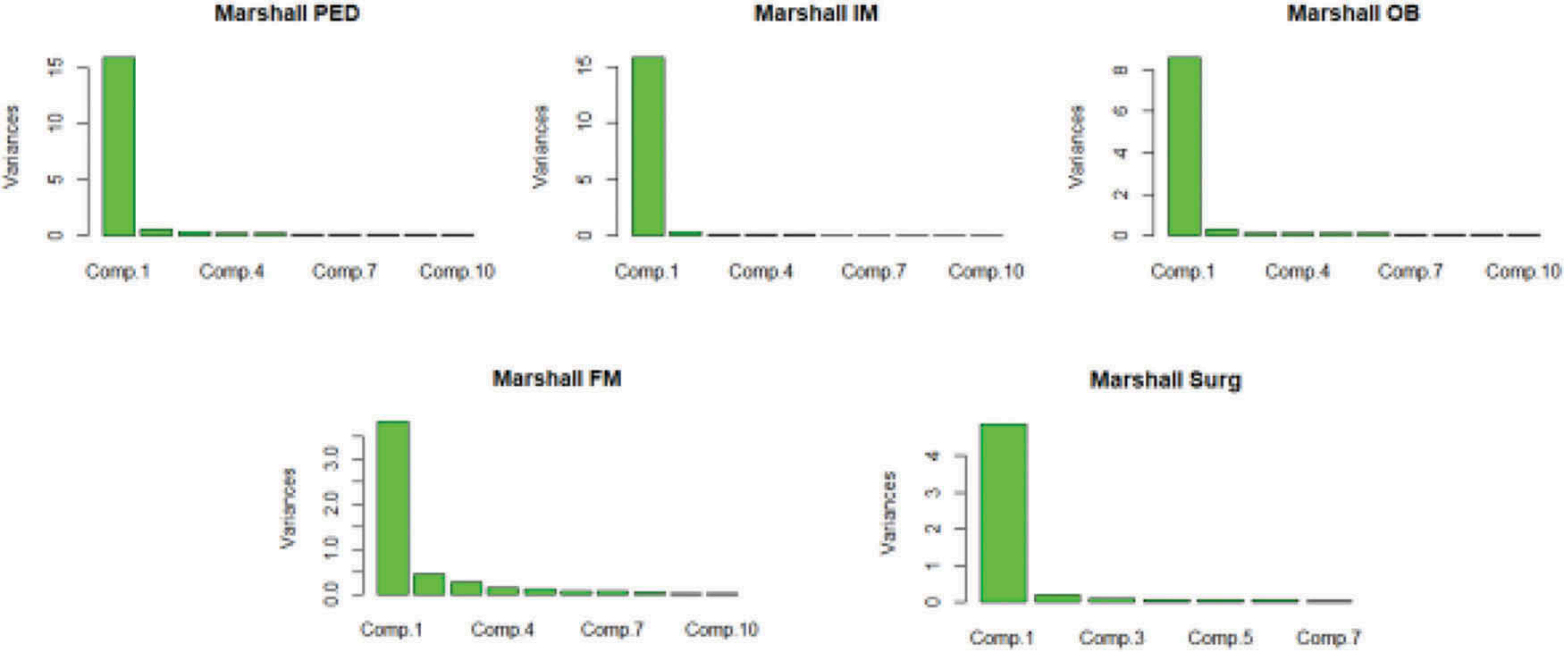
**Principal component analysis of Marshall Residency programs**. Note that for all programs, the first principal component contained the vast majority of information. In fact, for all programs, more than 95% of variance was contained within first 5 principal components. For FM, the distribution of significant variance went up to the 5^th^ component whereas for other programs, it was limited to the first 1 or 2.

When we performed principal component analysis, we found that > 95% of the variance could be explained by the first 5 principal components in all programs. In all but FM, almost all of the variance was contained within the first 2 principal components (Figure 4).

Although it was tempting to speculate that the longer questionnaires had greater correlation values, this was not necessarily the case. In fact, the Marshall Surgery program with only 7 Likert questions had the highest average correlations between the answers. Next highest at Marshall was the Psych questionnaire with 65 Likert questions. Conversely at UT, Psych had the lowest average correlation between answers to Likert questions while having the highest number of Likert questions within that institution.

## Discussion

The evaluation of post-graduate physician trainees in ACGME programs has undergone an infusion of rigor and structure during the past decades. Some of this can be attributed to the introduction of the six core competencies approximately 20 years ago [9].

While increased granularity of evaluation is clearly desirable, our data would suggest that we see diminishing returns. As demonstrated in Figure 3, one must speculate that if the answers to Likert questions correlate so highly with one another, there must be some degree of redundancy. Without proof, we would conjecture that the thoughtfulness of response might be compromised by a task perceived as onerous by the responding faculty member [10]. An alternative is that faculty, when evaluating the clinical performance of residents, simply don’t or aren’t able to discriminate between performance in core competencies. As an example, if the resident is perceived to be high performing, that may be generalized to all domains of the evaluation. In contrast, if the resident is perceived as poorly performing, that may also be generalized to all components of their evaluation. Certainly, the high degree of correlation between questions suggests that residents receive modest benefit in the identification of specific areas of competence that could be improved.

When we examined our data between the different institutions, it was apparent that the correlation coefficients varied more within the UT than Marshall programs, suggesting that not only were the correlations between answers to questions lower but the variability of said correlations was greater. This suggests that more information might be present in the UT evaluations; unfortunately, without a true gold standard it is difficult to confirm this. As we examine the specific Likert scale responses between departments, it was also clear that the scores given to the Marshall Psych residents were on average lower than that for the other core residency programs. This is evident despite the Likert scale being extended to 6 for some questions on the Psych form, whereas 5 was the upper limit for the other program questions. This discrepancy is almost certainly due to the Psych residency being new and our cadre of Psych residents limited to the PGY1 and 2 years for the academic year under consideration. It seems that the Likert questions alone for the Psychiatry form at Marshall would require in excess of one hour to complete. That said, it would be unlikely that faculty would uniformly fill such a form out with diligence and thoughtfulness. The IM questionnaires from both institutions as well as Psychiatry, FM and Surgery from UT each had nearly ½ that number of Likert questions and would also appear to be perceived as onerous. In contrast, Marshall Surgery and Marshall Family Medicine managed to evaluate their residents with much briefer forms.

Following the analysis presented in this manuscript, the authors have concluded that residency evaluation forms should be simplified. It would seem that an evaluation form focused on the six basic competencies along with an additional question or two regarding other relevant activities (e.g., teaching medical students [11]) would represent an improvement over the larger, unwieldy forms employed by some of our residency programs. We would also suggest that such forms be evaluated on an ongoing basis; although comparison over time is an appealing advantage for maintaining consistency, it may be more important to modify these forms to reflect the actual axes of evaluation that are performed by evaluators which may, in fact, change year to year.
